# MMP‐7 affects peritoneal ultrafiltration associated with elevated aquaporin‐1 expression via MAPK/ERK pathway in peritoneal mesothelial cells

**DOI:** 10.1111/jcmm.16697

**Published:** 2021-06-11

**Authors:** Yue Yin, Fen Zhang, Zhuojun Zheng, Zhiwen Xiao, Qiaomu Yang, Nirong Gong, Jia Zhou, Daming Zuo, Jun Ai

**Affiliations:** ^1^ Division of Nephrology State Key Laboratory of Organ Failure Research National Clinical Research Center of Kidney Disease Guangdong Provincial Key Laboratory of Renal Failure Research Guangzhou Regenerative Medicine and Health Guangdong Laboratory Nanfang Hospital Southern Medical University Guangzhou China; ^2^ Department of Medical Laboratory School of Laboratory Medicine and Biotechnology Southern Medical University Guangzhou China; ^3^ Department of Immunology School of Basic Medical Sciences Southern Medical University Guangzhou China; ^4^ Microbiome Medicine Center Zhujiang Hospital Southern Medical University Guangzhou China

**Keywords:** aquaporin‐1, extracellular signal‐regulated kinase, matrix metalloproteinases‐7, peritoneal membrane dysfunction, ultrafiltration failure

## Abstract

Peritoneal membrane dysfunction and the resulting ultrafiltration failure are the major disadvantages of long‐term peritoneal dialysis (PD). It becomes increasingly clear that mesothelial cells play a vital role in the pathophysiological changes of the peritoneal membrane. Matrix metalloproteinases (MMPs) function in the extracellular environment of cells and mediate extracellular matrix turnover during peritoneal membrane homeostasis. We showed here that dialysate MMP‐7 levels markedly increased in the patients with PD, and the elevated MMP‐7 level was negatively associated with peritoneal ultrafiltration volume. Interestingly, MMP‐7 could regulate the cell osmotic pressure and volume of human peritoneal mesothelial cells. Moreover, we provided the evidence that MMP‐7 activated mitogen‐activated protein kinases (MAPKs)‐extracellular signal‐regulated kinase 1/2 (ERK) pathway and subsequently promoted the expression of aquaporin‐1 (AQP‐1) resulting in the change of cell osmotic pressure. Using a specific inhibitor of ERK pathway abrogated the MMP‐7‐mediating AQP‐1 up‐regulation and cellular homeostasis. In summary, all the findings indicate that MMP‐7 could modulate the activity of peritoneal cavity during PD, and dialysate MMP‐7 might be a non‐invasive biomarker and an alternative therapeutic target for PD patients with ultrafiltration failure.

## 1 INTRODUCTION

Peritoneal dialysis (PD) is a process that provides patients not only with solute clearance but also with fluid removal, which is called ultrafiltration. Ultrafiltration failure remains a common complication of long‐term PD, especially in patients without residual renal function (RRF).^1^ Recurrent ultrafiltration failure in PD patients is closely associated with water retention, hypertension resistance,^2^ pulmonary oedema^3^ and acute or chronic congestive heart failure.^4^ Besides interstitial thickening and sclerosis caused by repeated infection or long‐term exposure to unphysiological dialysate, water channel proteins damaging in the peritoneal membrane is also an important factor for ultrafiltration failure.^5^ Aquaporin‐1 (AQP‐1), one of the water‐specific channel proteins distributed in the endothelium lining the peritoneal capillaries, facilitates the osmotic transport of water across the capillary endothelium, thereby playing an essential role in ultrafiltration during PD.^6^ Knocking out AQP‐1 gene in mice reduced osmotically driven water transport and approximately 50% of total ultrafiltration, while pharmacological agonist AqF026 enhanced this water transport.^7^ Lai et al had reported an inducible expression of AQP‐1 in human peritoneal mesothelial cells.^8^ Even though there was a significant enhancement of AQP‐1 biosynthesis upon exposure to osmotic agents such as glucose and mannitol^8^ in peritoneal endothelial cells, the role of AQP‐1 in maintaining the ultrafiltration of the peritoneal mesothelial cells is still not fully recognized.

Matrix metalloproteinases (MMPs), the family of secreted and membrane‐bound zinc‐dependent endopeptidases, are expressed in both developing and adult kidneys, and increasing evidence pointed out the function of MMPs in both normal physiology and abnormal pathology in the kidney.^9^ MMPs are increasingly known to cleave a wide variety of substrates, ranging from cell surface receptors and adhesion molecules to growth factors and cytokines.^9^, ^10^, ^11^ Therefore, MMPs promote cell proliferation, migration and differentiation and are also thought to play a significant role in cell apoptosis, angiogenesis, tissue repair and immune response.^10^, ^11^, ^12^ Among all the MMPs, MMP‐7 is the smallest secreted MMP with a broad substrate specificity, consisting of a pro‐peptide domain and a catalytic domain. It plays an important role in degrading the extracellular matrix and many other biological activities.^13^ Generally, the primary function of MMP‐7 is to break down the extracellular matrix by digesting casein, gelatines, fibronectin and proteoglycan. Such an ability of MMP‐7 acting on a broad spectrum of substrates indicates its role in controlling numerous biological processes such as tissue remodelling, apoptosis and inflammation.^13^, ^14^

MMP‐7 has been detected in the serum in patients with disease states, such as chronic inflammatory diseases, some epithelial‐derived tumours and some acute organic injuries.^15^, ^16^ A previous study showed that MMP‐7 is a downstream target gene of Wnt/β‐catenin signalling pathway and is involved in kidney fibrosis.^14^ Even though the increased serum MMP‐7 in chronic kidney disease (CKD) patients has proved to be acted as a non‐invasive biomarker of pre‐fibrotic signalling,^16^ and the urine MMP‐7 was a promising predictor for acute kidney injury severity,^13^ the distribution and the potential role of MMP‐7 in PD patients are still unknown. To test this, we conducted a cross‐sectional study in a cohort of 295 PD patients. We observed a higher concentration of both serum and dialysate MMP‐7 levels in these patients, and the increased MMP‐7 in the peritoneal dialysate was negatively associated with peritoneal ultrafiltration. In human peritoneal mesothelial cells, we found that MMP‐7 could regulate the cell osmotic pressure and volume. Furthermore, we provided the evidence that MMP‐7 activated mitogen‐activated protein kinases (MAPKs)‐extracellular signal‐regulated kinase 1/2 (ERK) pathway and subsequently promoted the expression of AQP‐1, resulting in the change of cell osmotic pressure. These data suggested the possibility of using MMP‐7 as a biomarker and an alternative therapeutic target for PD patients with ultrafiltration failure.

## 2 MATERIALS AND METHODS

### 2.1 Subjects

Healthy controls (HC, *n* = 20) and ESRD patients who had been undergoing PD for at least more than 1 month (*n* = 303) were involved in this cross‐sectional study from 1 June 2018 to 31 December 2018 in the Renal Department, Nanfang Hospital, Southern Medical University. All willing participants were included, and there were no exclusion criteria. The study protocol was approved by the research ethics committee of Nanfang Hospital, Southern Medical University (ethics number NFEC‐201809‐K1), and all participants provided written informed consent.

We collected blood samples and 24‐hour dialysate samples at the same visit point. Clinical data including age, sex, causes of ESRD and comorbidity conditions (hypertension, diabetic mellitus and hepatitis B virus infection) were recorded at the same time. Data of blood pressure, PD vintage, dialysate glucose concentration, 24‐hour PD ultrafiltrated volume, 24‐hour urine volume, weekly total KT/V, peritoneal equilibration test (PET) typed, serum levels of Cr, albumin, phosphorus and calcium, intact parathyroid hormone and blood haemoglobin were also collected and recorded. Information on patients’ medical history and medications was obtained from their medical records. Serum Cr, albumin, calcium and phosphate was measured using an automatic biochemical analyser (AU480; Olympus) in our hospital. Serum parathyroid hormone levels were measured by chemiluminescence assay (D‐68350; Roche Diagnostics). Haemoglobin was tested by a routine blood test analyser (XN9000; Sysmex). Conventional weekly total KT/V and PET types were measured by standard methods. MMP‐7 in serum, dialysate and cell culture supernatant were measured by ELISA kit (DY907; R&D Systems) and were measured in triplicate. The intra‐ and inter‐assay variability ranged between 3%‐6% and 2%‐8% based on blinded replicate samples from study patients, respectively.

### 2.2 Cell culture

Human peritoneal mesothelial cell (HMrSV5) was bought from Jennio Biotech and cultured in minimum essential medium (MEM) (Gibco) supplemented with 10% foetal bovine serum (FBS) (Gibco) and 100 U/ml penicillin‐streptomycin (Sangon Biotech Co., Ltd.) at the atmosphere of 37℃ in 5% CO_2_.

### 2.3 Reagents and antibodies

Recombinant human matrix metalloproteinases‐7 protein was obtained from AmyJet Scientific. The chemical inhibitors for the MAPK pathway (ie U0126, SB203580 and JNK‐IN‐8) were purchased from Selleck Chemicals. Antibodies (Abs) against phospho‐ERK, phospho‐JNK, phospho‐p38, phospho‐β‐catenin, phospho‐AKT, phospho‐mTOR, phospho‐p65, ERK, JNK, p38, β‐catenin, AKT, PI3K p110, PI3K p85 and mTOR, p65 were obtained from Cell Signaling Technology. Anti‐AQP‐1(AF5231) and Anti‐MMP‐7(AF0218) were brought from Affinity Biosciences LTD. HRP‐conjugated Goat Anti‐Rabbit IgG and HRP‐conjugated Goat Anti‐Mouse IgG were bought from Proteintech.

### 2.4 Lentiviral infection

HMrSV5 cells (1.5 × 10^6^ per 60 mm dish) were incubated with the suggested volume of lentivirus negative (GeneCopoeia, Ex‐NEG‐Lv201, iGeneBio) and lentivirus MMP‐7 (GeneCopoeia, LPP‐F0265‐Lv201‐100, iGeneBio). Cell transfection can be observed through GFP fluorescence under a fluorescence microscope. Then, the cells were selected by the culture medium including 2.5 nM puromycin.

### 2.5 siRNA‐mediated RNA interference

Control siRNA (sictrl) and AQP‐1‐targeting siRNAs (siAQP‐1) were purchased from RiboBio Biotechnology. HMrSV5 cells were transfected with the siRNA using the riboFECT CP (RiboBio) reagent according to the standard protocol and incubated for 36 hours before the next assay.

### 2.6 Extraction of membrane and cytoplasm protein fractions

We used the ProteinExt® mammalian membrane protein extraction kit purchased from TransGen Biotech to separate membrane and cytoplasm protein of cells. 5 × 10^6^ cells were washed twice with 1 ml of cold PBS and centrifuged for 3 minutes by 1,000 × g, and then, the supernatant was discarded. 750 μl of membrane protein extraction buffer I (MPEB I) was added to the cell precipitation, and the lysate was mixed well in vortex for 15 seconds. After the lysate was incubated in ice for 10 minutes, the mixture was centrifuged for 15 minutes by 16,000 × g in 4℃. The supernatant including cytoplasmic protein was carefully transferred into a clean EP tube. 150 μl MPEB I was added to the precipitation, and the precipitation was suspended again for 15 seconds, incubated in ice for 30 minutes. 300 μl MPEB I was added into the mixture, and then, the mixture was centrifuged for 15 minutes by 16,000 × g in 4℃. The supernatant including membrane protein was carefully collected into another EP tube. The extracted membrane and plasma protein can be used for further analysis.

### 2.7 Western blot

Protein samples lysed with RIPA buffer (50 mM Tris, 150 mM NaCl, and 1% Nonidet P‐40, pH 7.4) were separated on SDS‐polyacrylamide gels and then transferred onto polyvinylidene fluoride (PVDF) membranes (Millipore). The membranes were blocked by bovine serum albumin (BSA, 5%) for 1 hour at room temperature and incubated with indicated primary antibodies at 4℃ overnight. Subsequently, the membranes were stained with horseradish peroxidase (HRP)–conjugated corresponding secondary antibody.

### 2.8 RNA isolation and real‐time PCR

Isolated hepatocytes were homogenized in 1 ml TRIzol (Thermo Fisher Scientific), and total RNA was extracted based on the manufacturer's instruction. Total RNA was synthesized to cDNA using TransScript Fly First‐Stand cDNA Synthesis SuperMix (TransGen). Real‐time PCR SYBR Green technology was applied to quantify reverse‐transcribed mRNAs on an ABI Prism 7500 Sequence Detection System (Thermo Fisher Scientific), according to the following program. The levels of the target gene were normalized with respect to GAPDH gene expression.

### 2.9 Immunofluorescence assay

HMrSV5 cells were seeded on a 3.5 mm confocal dish and fixed at room temperature using 4% paraformaldehyde, and the cells’ membrane was broken with 0.5% Triton‐X100 or not. AQP‐1 proteins were stained and examined with confocal microscopy equipped with analytical software. The image was observed in three fields for each sample, and the mean fluorescence intensity of GFP and AQP‐1 was measured by ImageJ software.

### 2.10 Cell volume analysis

HMrSV5 cells were trypsinized and resuspended in different mediums (normal saline (NS) or 4.25% glucose peritoneal dialysis). The cell diameter was measured using the Scepter 2.0 cell counter (Merck Millipore) as previously described.^17^ After the resultant cell suspensions had been transferred to 5 ml EP tubes, the cells were counted with a Scepter 2.0 cell counter equipped with a sensor tip, according to the manufacturer's recommendation. Cell counts and cell size distributions were shown as histograms on the monitor of the Scepter 2.0 cell counter, and these data were analysed with the Scepter 2.0 Software Pro computer software.

### 2.11 Statistical analysis

Stata 15 software was used for the statistical analyses. The continuous variables were summarized as means and standard deviations (SD), and the categorical variables were listed as the number of cases and percentages. The comparison of continuous variables was done through the use of Student's *t* test, and the categorical variables were assessed with either a chi‐square test or a Fisher's exact test in patients with or without renal residual function (RRF). Unadjusted and adjusted (with age, sex, history of diabetic mellitus, RRF, peritoneal Kt/V and dialysate glucose concentration) linear regression analyses were performed to determine the relationships between MMP‐7 and peritoneal ultrafiltrated volume. *P* < 0.05 was considered to be statistical significance.

## 3 RESULTS

### 3.1 High‐expressed MMP‐7 was associated with peritoneal ultrafiltration in PD patients

**FIGURE 1:**
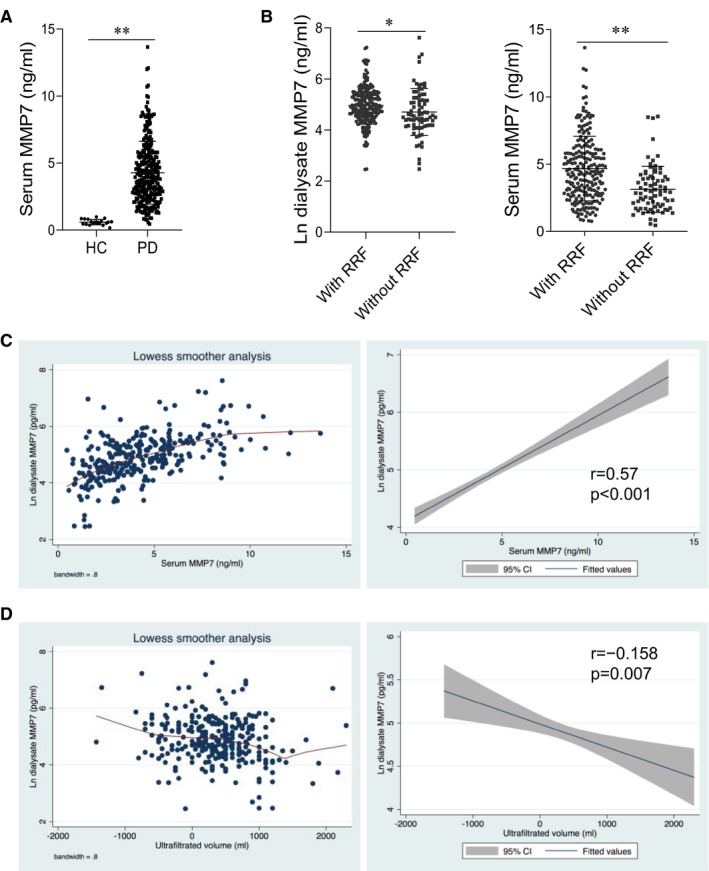
MMP‐7 might be an independent risk factor for ultrafiltration failure in peritoneal dialysis. (a) The levels of MMP‐7 in the serum of healthy control (HC) group compared with peritoneal dialysis patients. (b) The levels of MMP‐7 in the dialysate (pg/ml) and serum (ng/ml) of patients with residual renal function (RRF) compared with patients without RRF. (c) Linear regression analysis of the relationship between dialysate MMP‐7 and serum MMP‐7 in PD patients. (d) Linear regression analysis of the association between dialysate MMP‐7 and peritoneal ultrafiltration volume in ESRD patients. *P < 0.05, **P < 0.01, one of the three independent experiments is shown

Because 8 patients had failed to examine MMP‐7, 295 patients entered the analysis at last. Among them, the mean age was 43.5 ± 14.6 years, 54.6% were men, and the median dialysis vintage was 3 (1‐18) months. Detailed demographic, clinical and laboratory characteristics between participants with RRF (*n* = 219) and without RRF (*n* = 76) are shown in Table 1. Patients without RRF had lower body mass index (BMI), blood pressure, eGFR and longer dialysis vintage than those with RRF. A significantly increased level of MMP‐7 in serum was observed in patients with peritoneal dialysis compared with healthy controls (4.28 ± 2.34 ng/ml vs 0.50 ± 0.29 ng/ml, *P* < 0.0001; Figure 1a and Table S1). Interestingly, both serum and dialysate MMP‐7 levels were lower in the patients without RRF than those with RRF (Figure 1b). Because the dialysate MMP‐7 presented a tightly linear association with serum MMP‐7 (*r* = 0.57, *P* < 0.001; Figure 1c), we mainly analysed the dialysate MMP‐7. As shown in Figure 1d and Table 2, the dialysate MMP‐7 level was negatively associated with peritoneal UF volume (*P* = 0.007). When adjusted with demographics (age, sex), history of diabetic mellitus, RRF, peritoneal dialysis adequacy (peritoneal KT/V) and dialysate glucose concentration, the dialysate MMP‐7 still significantly correlated to peritoneal UF volume (β = −76.1, *P* = 0.039; Table 2). Taken together, these data indicate a negative association of the dialysate MMP‐7 level with peritoneal UF volume in PD patients.

**TABLE 1:** Clinical characteristics by residual renal function

Characteristics	All (*n* = 295)	With RRF (*n* = 219)	Without RRF (*n* = 76)	*p* value
Age, yr	43.5 ± 14.6	43.2 ± 14.5	44.3 ± 15.0	0.555
Male, %	54.6	57.7	46.1	0.078
BMI	21.6 ± 3.4	21.9 ± 3.6	20.9 ± 2.6	0.021
History of DM, n(%)	68 (23.1)	58(26.5)	10 (13.2)	
Clinical data				0.018
SBP, mmHg	147.9 ± 23.4	150.0 ± 22.8	142.2 ± 24.2	0.013
DBP, mmHg	93.6 ± 15.1	97.5 ± 14.5	90.8 ± 16.5	0.061
Peritoneal dialysis vintage, mo	3 (1‐18)	2 (1‐5)	34 (16‐35)	<0.001
Dialysate GLU concentration, %	1.7 ± 0.3	1.6 ± 0.2	2.0 ± 0.3	<0.001
Dialysate UF volume, ml	350 (40‐700)	250 (−150‐500)	775 (500‐1050)	<0.001
Laboratory examination				
Serum creatinine, mg/dl	10.8 ± 3.9	9.84 ± 3.5	13.7 ± 3.4	<0.001
eGFR^a^, ml/min	5.31 ± 2.48	5.92 ± 2.54	3.52 ± 0.96	<0.001
Serum albumin, g/L	36.7 ± 4.5	36.8 ± 4.5	36.5 ± 4.6	0.601
Blood haemoglobin, g/L	113.5 ± 23.9	114.6 ± 24.9	110.3 ± 20.7	0.185
Serum calcium, mmol/L	2.32 ± 0.23	2.31 ± 0.22	2.32 ± 0.25	0.854
Serum phosphate, mmol/L	1.75 ± 0.59	1.69 ± 0.57	1.89 ± 0.64	0.009
Ln Serum PTH, pg/ml	5.78 ± 0.96	5.68 ± 0.84	6.06 ± 1.19	0.003
Serum MMP‐7, ng/ml	4.28 ± 2.34	4.66 ± 2.40	3.12 ± 1.73	<0.001
Ln dialysate MMP‐7, pg/ml	3.81 ± 1.98	3.38 ± 1.73	6.53 ± 0.98	<0.001
Indices of dialysis adequacy				
Dialysate weekly KT/V	1.73 ± 0.66	1.67 ± 0.73	1.91 ± 0.32	0.005
RRF weekly KT/V	0.80 ± 0.51	0.80 ± 0.51	—	—
Total weekly KT/V	2.33 ± 0.79	2.47 ± 0.86	1.92 ± 0.32	<0.001

Note

Continuous variables were expressed as mean ±SD or median (25th percentile‐75th percentile). Categorical variables were expressed as n (%). Weekly KT/V and UF volume (ml/24 h) were calculated by formulas mentioned before.

Abbreviations: RRF, residual renal function; BMI, body mass index; DM, diabetic mellitus; SBP, systolic blood pressure; DBP, diastolic blood pressure; GLU, glucose; UF, ultrafiltrated; eGFR, evaluated glomerular filtrate rate; PTH, parathyroid hormone; MMP‐7, matrix metalloproteinase‐7.

^a^ eGFR was determined by the Chronic Kidney Disease Epidemiology Collaboration Equation (2009).

^b^ P for comparisons between with and without RRF groups by t test and chi‐square tests for continuous and categorical variables, respectively.

**TABLE 2:** Liner Regression of peritoneal UF volume with dialysate MMP‐7

Variables	Unadjusted		Adjusted^a^	
	β (95% CI)	*p* value	β (95%CI)	*p* value
Age, yr	0.96 (−3.55‐5.48)	0.675	−0.22 (−4.52‐4.08)	0.920
Male	−183.1 (−313.8‐−52.3) −183.1 (−313.8‐−52.3)	0.0060.006	−144.3 (−265.4‐−23.3)	0.020
DM	−185.5 (−340.6‐−30.4)	0.019	−51.7 (−194.6‐91.3)	0.477
With RRF	−644.2 (−775.5‐−512.9)	<0.001	−441.3 (−6013‐−281.2)	<0.001
Peritoneal KT/V	220.1 (123.3‐316.8)	<0.001	101.1 (8.21‐194.1)	0.033
Dialysate GLUC	787.8 (585.4‐990.3)	<0.001	323.0 (87.7‐558.3)	0.007
Ln Dialysate MMP‐7, pg/ml	−112.3 (−193.7‐31.1)	0.007	−76.1 (−148.3‐−3.93)	**0.039**

Note

Weekly KT/V and UF volume (ml/24 hours) of PD patients were calculated by formulas mentioned before.

Abbreviations: UF, ultrafiltrated; MMP‐7, matrix metalloproteinase‐7; BMI, body mass index; DM, diabetic mellitus; RRF, residual renal function, defined as 24 hours of urine volume >100 ml. GLUC, glucose concentration.

^a^ Adjusted with demographics (age, sex), with DM, with RRF, peritoneal dialysis adequacy (peritoneal KT/V) and dialysate GLUC.

### 3.2 High glucose induced the expression of MMP‐7 in peritoneal mesothelial cells

**FIGURE 2:**
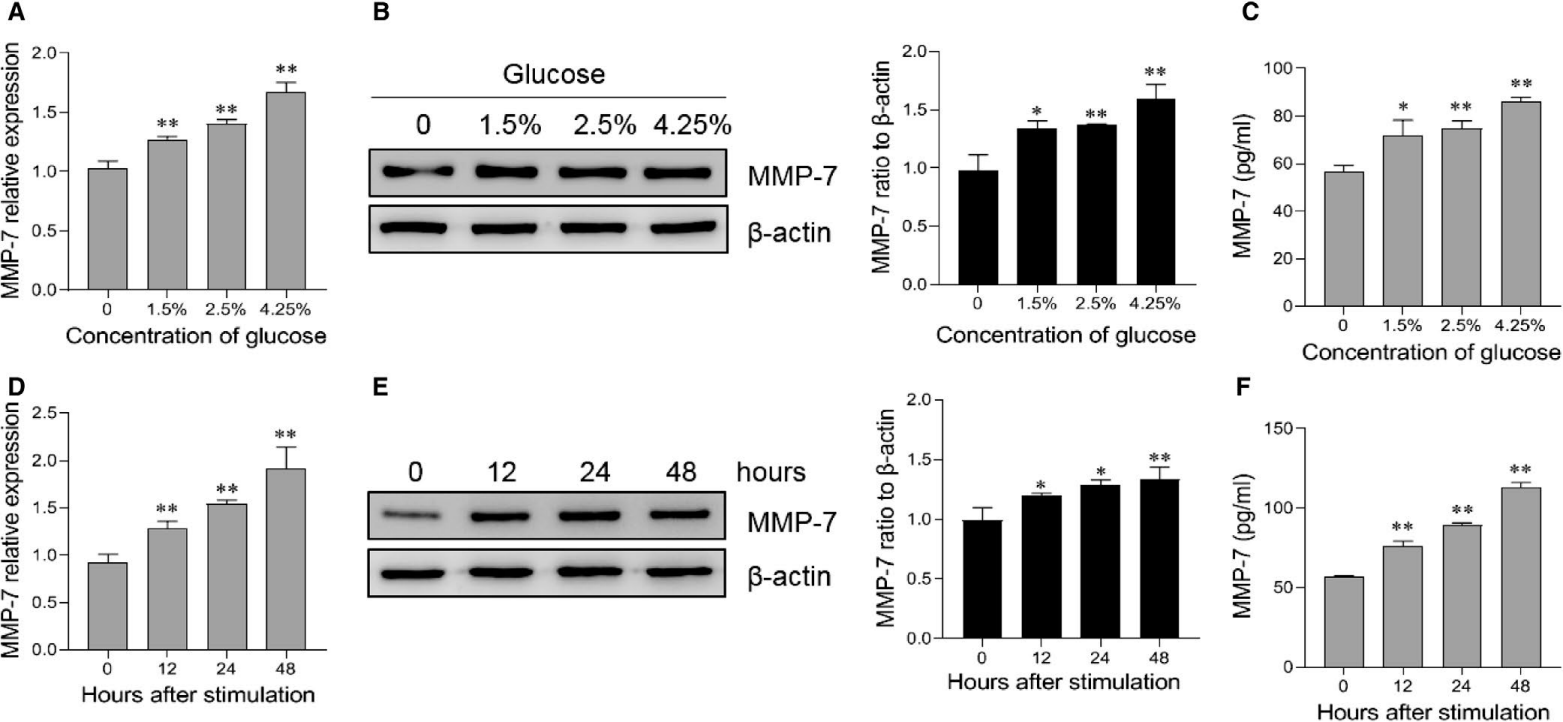
High glucose induced the expression of MMP‐7 in peritoneal mesothelial cells. (a, b) Peritoneal mesothelial cells were seeded in the 6‐well plate (1 × 10^6^ cells/well) and then stimulated with different concentrations of glucose for 24 hours. The mRNA level of MMP‐7 was detected through quantitative RT‐PCR (a), the protein level of MMP‐7 in the cells was detected by Western blotting analysis (b), and the production of MMP‐7 in the culture medium was detected by ELISA (c). (d‐e) Cells were incubated in dialysate with 4.25% glucose for indicated time points. The mRNA level of MMP‐7 was detected through quantitative RT‐PCR (d), the protein level of MMP‐7 in the cells was detected by Western blotting analysis (e), and the production of MMP‐7 in the culture medium was detected by ELISA (F). *P < 0.05, **P < 0.01, one of the three independent experiments is shown

Since glucose‐based peritoneal dialysis solutions are still predominantly used dialysate in PD patients in China, and the exposure to glucose might correlate to some unphysiological changes,^18^ we then examined the MMP‐7 expression under glucose exposure in human peritoneal mesothelial cells. As expected, enhanced MMP‐7 expression in HMrSV5 cells was also seen when titrated amounts of glucose were added (Figure 2a). The protein expression of MMP‐7 was strongly induced by high glucose determined by Western blotting (Figure 2b) and by ELISA (Figure 2c). Also, the result showed that glucose increased the MMP‐7 expression in a time‐dependent manner (Figure 2d‐f). These data suggest that high glucose concentration up‐regulates the expression of MMP‐7 in peritoneal mesothelial cells.

### 3.3 Recombinant MMP‐7 protein altered cell volume and enhanced AQP‐1 expression in peritoneal mesothelial cells

**FIGURE 3:**
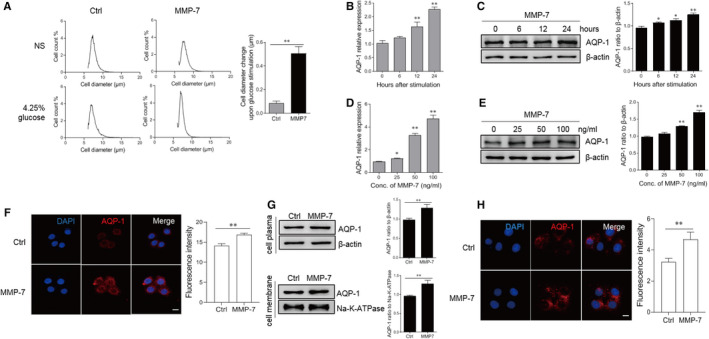
Recombinant MMP‐7 protein up‐regulated cell volume and enhanced aquaporin‐1 expression in peritoneal mesothelial cells. The HMrSV5 cells were seeded in the 6‐well plate (1 × 10^6^ cells/well). (a) Cells were stimulated with MMP‐7 protein for 12 hours, followed by incubating in normal saline (NS) or dialysate with 4.25% glucose for 1 minute. The cell diameter of peritoneal mesothelial cells was evaluated by the Scepter 2.0 cell counter. The changes of cell diameter after glucose stimulation, with or without MMP‐7 incubation, were calculated. (b, c) The cells were treated with 50 ng/ml MMP‐7 for different time points. The mRNA level of AQP‐1 was detected by quantitative RT‐PCR (b), and the protein levels of AQP‐1 were detected by Western blotting (c). (d, e) The cells were treated with different concentrations of MMP‐7 for different time points. The mRNA level of AQP‐1 was detected by quantitative RT‐PCR (d), and the protein levels of AQP‐1 were detected by Western blotting (e). (f) The cells were treated with indicated 50 ng/ml MMP‐7 for 12 hours. The cells were then permeabilized by Triton‐X 100, and immunofluorescence assay was used to detect the expression of AQP‐1 (red, with DAPI‐stained blue nuclei). (g, h) The cells were treated with the indicated 50 ng/ml MMP‐7 for 12 hours. The expression of AQP‐1 in the HMrSV5 cell membrane was evaluated by Western blotting (g) and immunofluorescence assay (H). *P < 0.05, **P < 0.01, one of the three independent experiments is shown

During peritoneal dialysis, the fluid pressure interacts with osmotic pressure to regulate cell volumes. We next investigated whether MMP‐7 treatment affected the volume of peritoneal mesothelial cells. Compared to the control group, treatment with MMP‐7 recombinant protein significantly altered the volume of HMrSV5 cells, indicating that MMP‐7 modulated the cellular volume for maintaining intracellular osmotic pressure (Figure 3a). AQP‐1, the universal water channel, is responsible for the rapid response of cell volume.^19^ We then addressed whether MMP‐7 affects the expression of AQP‐1 in HMrSV5 cells. We observed that MMP‐7‐treated cells displayed increased AQP‐1 expression compared to the control cells, as indicated by real‐time PCR and immunoblotting assay (Figure 3b‐e). Meanwhile, we assessed the protein expression level of AQP‐1 by immunofluorescence (Figure 3f). In addition, the immunoblotting analysis determined that MMP‐7 recombinant protein treatment enhanced the AQP‐1 expression in both cell membrane and cytoplasm (Figure 3g). We also tested the protein expression level of AQP‐1 in the cell membrane by immunofluorescence without using Triton‐X 100 (Figure 3h). The results indicate that extracellular MMP‐7 promotes the cell volume and AQP‐1 expression in peritoneal mesothelial cells.

### 3.4 Ectopic expression of MMP‐7 altered cell volume and enhanced AQP‐1 expression in peritoneal mesothelial cells

**FIGURE 4:**
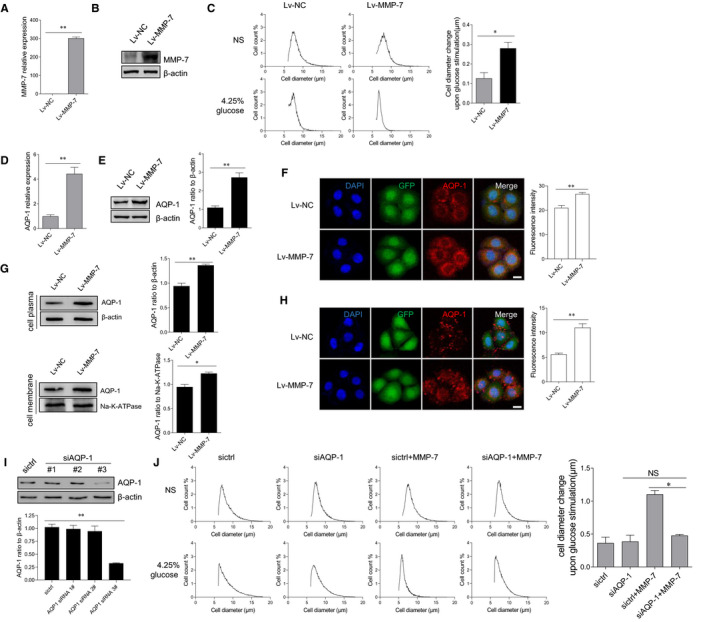
Ectopic expression of MMP‐7 enhanced AQP‐1 expression and then altered cell volume in peritoneal mesothelial cells. HMrSV5 cells were infected with lentivirus expressing MMP‐7. (a, b) The expression of MMP‐7 in the lentivirus‐transfected cells was determined by quantitative RT‐PCR (a) and Western blotting analysis (b). (c) The MMP‐7‐overexpressing cells (Lv‐MMP‐7) and control cells (Lv‐NC) were incubated in NS or dialysate with 4.25% glucose for 1 minute. The cell diameter of peritoneal mesothelial cells was evaluated by the Scepter 2.0 cell counter. The changes of cell diameter after glucose stimulation were calculated. (d‐f) The mRNA level of AQP‐1 was detected by quantitative RT‐PCR (d), and the protein expression of AQP‐1 was assessed by Western blotting assay (e). Besides, the cells were permeabilized by Triton‐X 100 and immunofluorescence assay was used to detect the expression of AQP‐1 (f). (g, h) The cells were treated with the indicated 50 ng/ml MMP‐7 for 12 hours. The expression of AQP‐1 in the HMrSV5 cell membrane was evaluated by Western blotting (g) and immunofluorescence assay (h), HMrSV5 cells were transfected with AQP‐1‐targeting siRNAs for 36 hours. The expression level of AQP‐1 protein was assayed by immunoblotting. The #3 siRNA against AQP‐1 was selected for the further analysis. (I) After transfecting with AQP‐1‐targeting siRNAs for 36 hours, HMrSV5 cells were incubated with 100 ng/ml MMP‐7 for another 24 hours. Cell diameter of the peritoneal mesothelial cells was evaluated by the Scepter 2.0 cell counter (J). *P < 0.05, **P < 0.01, one of the three independent experiments is shown

As known, MMPs have a novel proteolytic role in vivo.^20^ We, therefore, raised a question of whether ectopic expression of MMP‐7 influences the cellular volume in peritoneal mesothelial cells. The lentiviral expression system was employed for the overexpression of MMP‐7 in HMrSV5 cells (Figure 4a‐b). Overexpression of MMP‐7 in peritoneal mesothelial cells strongly enhanced the volume of HMrSV5 cells (Figure 4c). Meanwhile, enhanced AQP‐1 expression was exhibited in the MMP‐7‐overexpressed cells compared to that in the control cells, as indicated by real‐time PCR, immunoblotting assay (Figure 4d‐e) and immunofluorescence (Figure 4f). Furthermore, both cell membrane and cytoplasm expression of AQP‐1 were highly expressed (Figure 4g). We also got a similar result of the protein expression level of AQP‐1 in the cell membrane by immunofluorescence without Triton‐X 100 permeabilization (Figure 4h). To further verify whether the increase of cell diameter after MMP‐7 treatment is dependent on AQP‐1, we used siRNA to knock‐down AQP‐1 protein in HMRSV5 cells (Figure 4i). The result showed the cell volume was comparable between the cells treated with or without MMP‐7 when AQP‐1 expression in cells was silenced (Figure 4j). The results suggested that ectopic expression of MMP‐7 enhanced AQP‐1 expression and then altered cell volume in peritoneal mesothelial cells.

### 3.5 MMP‐7‐mediated ERK signalling activation was responsible for the enhancement activity of MMP‐7 on cellular volume in peritoneal mesothelial cells

**FIGURE 5:**
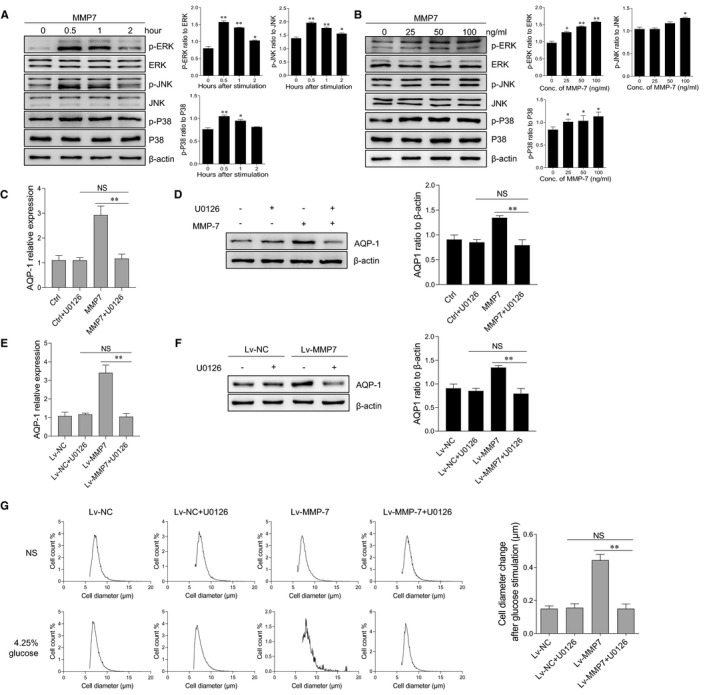
MMP‐7‐mediated ERK signalling activation is responsible for the enhancement activity of MMP‐7 on cellular osmotic pressure in peritoneal mesothelial cells. The HMrSV5 cells were seeded in the 6‐well plate (1 × 10^6^ cells/well). (a) HMrSV5 cells were stimulated with 100 ng/ml MMP‐7 protein for indicated time points, and the phosphorylation level of ERK, JNK and p38 was detected by Western blotting. (b) HMrSV5 cells were treated with different concentrations of MMP‐7 protein for 1 hour, and the phosphorylation level of ERK, JNK and p38 was detected by Western blotting. (c, d) After pre‐incubation with U0126 for 2 hours, HMrSV5 cells were stimulated by 100 ng/ml MMP‐7 protein for 24 hours. The mRNA and protein expression level of AQP‐1 was detected by quantitative RT‐PCR (C) and Western blotting analysis (d), respectively. (e, f) The HMrSV5 cells were infected with negative control lentivirus and MMP‐7‐overexpressed lentivirus and incubated with U0126 for another 24 hours. The mRNA and protein expression level of AQP‐1 was detected by quantitative RT‐PCR (e) and Western blotting analysis (f), respectively. (g, h) After pre‐incubation with U0126 for 2 hours, the MMP‐7‐overexpressed HMrSV5 cells were incubated in NS or dialysate with 4.25% glucose for 1 minute. The cell diameter of peritoneal mesothelial cells was evaluated by the Scepter 2.0 cell counter (g). The changes of cell diameter after glucose stimulation were calculated (h). *P* < 0.05, **P < 0.01, NS, not significant, one of the three independent experiments is shown

After determination of the effect of MMP‐7 in up‐regulating cell volume and AQP‐1 expression, we next moved on to elucidate the underlying mechanism. So far, multiple signalling pathways were reported to be involved in the activation of MMPs, such as MAPKs, NF‐κB, PI3K/AKT and Wnt/β‐catenin signalling pathway. We, therefore, sought to determine whether the MAPK members (ie ERK, p38 and JNK) were activated in HMrSV5 cells upon MMP‐7 treatment. The level of ERK phosphorylation was markedly enhanced after stimulation with MMP‐7 recombinant protein treatment, while the phosphorylation of p38 and JNK was slightly increased in the cell treated with MMP‐7 protein compared to the control cells (Figure 5a). Moreover, MMP‐7 treatment enhanced the phosphorylation of MAPKs in a dose‐dependent manner (Figure 5b). However, the activation of NF‐κB, PI3K/AKT and Wnt/β‐catenin signalling pathways were not affected by MMP‐7 treatment in peritoneal mesothelial cells (Figure S1).

To validate the impact of ERK activation on MMP‐7 activity, HMrSV5 cells were treated with U0126 to block ERK activation prior to incubation with MMP‐7. After ERK inhibition, MMP‐7‐mediated enhancement of mRNA and protein level of AQP‐1 was significantly inhibited (Figure 5c‐f). However, inhibition of p38 and JNK MAPK activation did not influence the up‐regulated expression of AQP‐1 by MMP‐7 protein treatment (Figure S2). Moreover, the cell volume and AQP‐1 expression were comparable between MMP‐7 overexpressed and control cells after ERK pathway inhibition (Figure 5g,h). Taken together, these data indicate that blockade of ERK activation abolishes the enhancement activity of MMP‐7 on cell volume and AQP‐1 expression in peritoneal mesothelial cells.

## 4 DISCUSSION

MMP‐7 is reactivated in a host of disease states, including cancer and chronic kidney disease. In this study, we had demonstrated that MMP‐7 was markedly expressed in PD patients and negatively associated with peritoneal ultrafiltration. Interestingly, the data revealed the high glucose‐induced MMP‐7 expression from peritoneal mesothelial cells. Most importantly, MMP‐7 could up‐regulate the cell volume of peritoneal mesothelial cells by activating the ERK pathway and increasing the expression of AQP‐1. These data presented the important role of MMP‐7 in peritoneal ultrafiltration.

Low level of MMP‐7 is expressed in normal healthy people. However, it is markedly induced in human and animal models of CKD and AKI. Many evidence suggests that the MMP‐7 level could serve as a non‐invasive biomarker for predicting AKI prognosis and monitoring CKD progression.^21^, ^22^ PD is a technique that exposes the patient to glucose, which may result in structural and functional damage of the peritoneal membrane.^18^ Of note, our study showed that MMP‐7 was significantly increased in the ESRD patients receiving PD. Peritoneal dialysate, which is continuously stayed in the peritoneal and renewed 4 times per day, is a major way for solute and fluid clearance in PD patients.^23^ In our cohort study, we did confirm the marked expression of MMP‐7 in PD patients, not only in the serum but also in the dialysate, and the dialysate MMP‐7 presented the quite well linear association with serum MMP‐7. Furthermore, after detailed analyses, we found that dialysate MMP‐7 was negatively associated with peritoneal ultrafiltration. These data indicated that MMP‐7 might affect the function of the peritoneal membrane, which might play a new pathophysiological role in PD ultrafiltration.

During peritoneal dialysis, peritoneal mesothelial cells are exposed repeatedly to a non‐physiological hypertonic environment. Longer exposure to the hypertonic medium was associated with partial recovery of the reduced mesothelial cell volume.^24^ Thus, the cells exposed to a medium supplemented with glucose might be accompanied by the reduction in cell volume. Interestingly, we observed an elevated expression and secretion of MMP‐7 from peritoneal mesothelial cells when exposed to high glucose. Most importantly, we determined that MMP‐7 could up‐regulate the cell volume of mesothelial cells. Cell volume regulation is one of the fundamental homeostasis of the cell that is associated with various cellular behaviours and functions.^25^, ^26^ Additionally, the disorder of cell volume homeostasis has been reported to be linked to the pathogenesis and progression of several diseases.^27^ After the mesothelial injury, mesothelial cells undergo tissue recovery. According to Nagai et al, the peritoneal mesothelial cells changed their morphology from a flattened shape to a cuboidal one prior to initiating migration to repair injured sites.^28^ MMP‐7 can regulate a diverse array of biological processes, such as podocyte dysfunction, cell proliferation, apoptosis and epithelial‐to‐mesenchymal transition (EMT).^22^ The results of our study revealed a new function of MMP‐7 in modulating cellular volume, which also expanded our understanding of the role of MMP‐7 in peritoneal ultrafiltration.

AQP‐1 confers a very high osmotic permeability and allows for rapid cell volume regulation in response to isosmotic solutions. It has been reported that hyperosmotic stress had a dose‐dependent effect on the regulatory control of cell volume.^29^ Actually, knock‐down of AQP‐1 in cells could induce changes in cell shape and F‐actin organization. It should be noted that the integrity of the F‐actin cytoskeleton is critically dependent on cell volume changes.^30^ Therefore, the interaction between the cytoskeleton and the water channels plays a critical role in the regulation of cell volume. A previous study determined that MMPs are implicated in the reorganization of the actin cytoskeleton.^31^, ^32^ In human trabecular meshwork cells, the alteration of actin cytoskeletal integrity is associated with MMP‐2 activation.^31^ Chintala et al found that disruption of the actin cytoskeleton, leading to cell shape changes, could suppress the MMP‐9 activation in human gliomas.^33^ Notably, MMPs produced by the peritoneal membrane have the capacity to degrade the extracellular matrix of the surrounding stroma. Therefore, the MMPs might be involved in the peritoneal membrane injury.^34^, ^35^ In our study, MMP‐7 significantly up‐regulated the expression of AQP‐1 accompanied by changes in cell volume. It is possible that MMP‐7 modulated the crosstalk between the activity of water channels and the stability of the cell cytoskeleton, and further investigation is warranted.

A great number of experimental data pointed out that the expression of MMPs is transcriptionally regulated by different pathways. In turn, MMPs participate in the activation of several pro‐inflammatory signalling pathways. Activation of the Wnt/β‐catenin signalling pathway has been suggested to regulate the expression of MMPs in many cells.^14^, ^36^ Notably, some types of MMPs, such as MMP‐2 and MMP‐9, are direct transcription targets of the Wnt/β‐catenin signalling pathway.^37^ MMP‐1 promotes the expression of the vascular endothelial growth factor receptor 2 (VEGFR2) via activating the transcription factor NF‐κB in endothelial cells.^38^ Similarly, the presence of MMP‐7 diminished the suppressive effect of sVEGFR‐1 on VEGF‐induced phosphorylation of VEGFR2 in human umbilical vein endothelial cells (HUVECs).^39^ Exogenously added MMP‐2 triggers the oxidized LDLs (oxLDLs), which induces the activation of both sphingomyelin/ceramide pathway and ERK pathway and DNA synthesis.^40^ Our current study provided evidence that MMP‐7 enhanced the activation of the ERK pathway in peritoneal mesothelial cells. Importantly, the inhibitor of ERK U0126 attenuated MMP‐7 inducing AQP‐1 production, which suggested that MMP‐7 enhanced the cellular osmotic pressure by activating the ERK pathway. Indeed, activation of ERK pathway is essential for cell growth, differentiation and integrin expression.^41^ Low et al observed that integrin is a family of multifunctional protein with functions in maintenance and regulation of cell volume.^42^ It is of interest to demonstrate whether MMP‐7 modulate cell volume through the ERK integrin axis, which is worth further research.

In summary, increased dialysate MMP‐7 level was negatively associated with peritoneal ultrafiltration in PD patients, and the MMP‐7 could up‐regulate cell volume by activating the ERK signalling pathway and subsequently enhancing the expression of AQP‐1 in mesothelial cells. Our findings provide an initial mechanistic insight of water re‐absorption and the potential MMP‐7‐targeted therapy in PD ultrafiltration failure.

## CONFLICT OF INTEREST

The authors have no conflicts of interest to declare.

## AUTHOR CONTRIBUTION

Yue Yin: Formal analysis (supporting); Methodology (supporting); Writing‐original draft (supporting). Feng Zhang: Data curation (equal); Methodology (equal); Writing‐original draft (equal). Zhoujun Zheng: Data curation (equal); Methodology (equal). Zhiwen Xiao: Methodology (supporting). Qiaomu Yang: Formal analysis (supporting); Methodology (supporting). Nirong Gong: Data curation (supporting); Formal analysis (supporting). Jia Zhou: Formal analysis (supporting). Daming Zuo: Conceptualization (equal); Investigation (equal); Writing‐review & editing (equal). Jun Ai: Conceptualization (equal); Formal analysis (equal); Funding acquisition (equal); Writing‐review & editing (equal).

## Supporting information

Supplementary Material

Supplementary Material

## Data Availability

The data used to support the findings of this study are included within the article.
