# Supplementary material for: MMP‐7 affects peritoneal ultrafiltration associated with elevated aquaporin‐1 expression via MAPK/ERK pathway in peritoneal mesothelial cells
Source: J Cell Mol Med. 2021 Jun 11;25(14):6887–98. doi: 10.1111/jcmm.16697 (PMC8278116; doi:10.1111/jcmm.16697)
Supplement: Supplementary file 1 — Supplementary Material [file JCMM-25-6887-s002.docx]

**Supplementary Table and Figures**

**Table S1 The comparison between PD patients and healthy controls**

| Variable | PD patients | Healthy Control | p ^a^ |
| --- | --- | --- | --- |
| n  Age (y)  Male [n(%)]  Serum MMP-7 (ng/ml) | 20  41.7 ± 8.6  10 (50.0)  0.50 ± 0.29 | 295  43.5 ± 14.6  161 (54.6)  4.28 ± 2.34 | -  0.542  0.304  <0.001 |

Continuous variables were expressed as mean ± SD or median (25th percentile-75th percentile). Categorical variables were expressed as n (%). PD, peritoneal dialysis. MMP-7, matrix metalloproteinase-7.

^a^. p for comparisons between PD patients and Healthy Control groups by t test and chi-square tests for continuous and categorical variables, respectively.


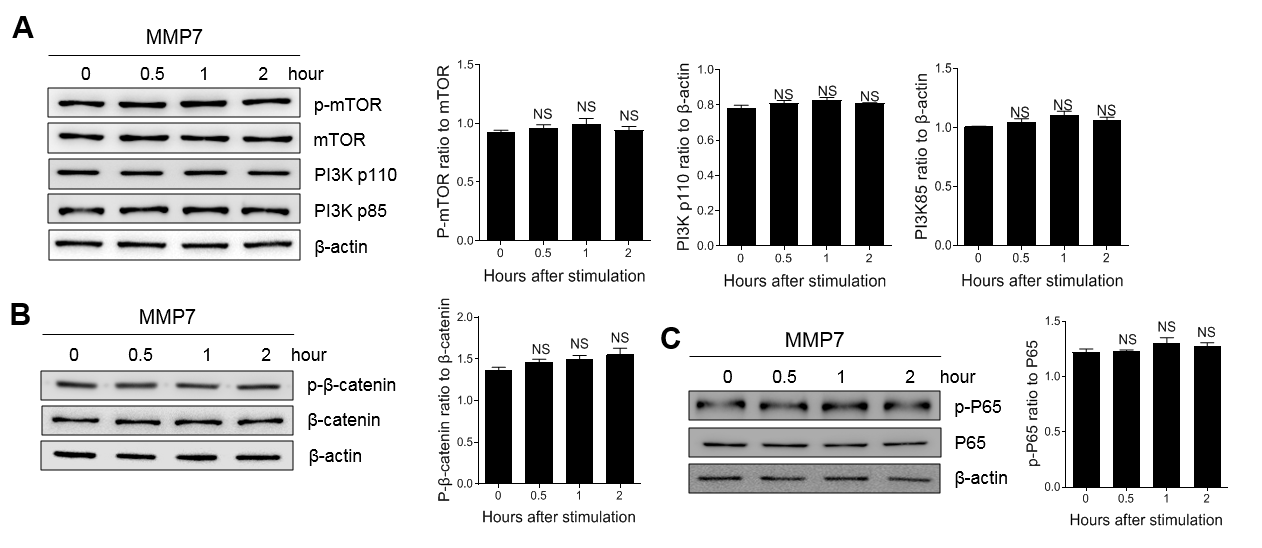


**Figure S1 The activation of NF-κB, PI3K/AKT, and Wnt/β-catenin signaling pathways in peritoneal mesothelial cells under stimulation by recombinant MMP-7 protein**

The HMrSV5 cells were stimulated with MMP-7 protein (100 ng/ml) for indicated time point. The activation of PI3K/Akt (A), Wnt/β- Catenin (B) and NF-κB (C) signaling pathway were evaluated by western blotting analysis. NS, not significant, compared to the control group. One of the three independent experiments is shown.


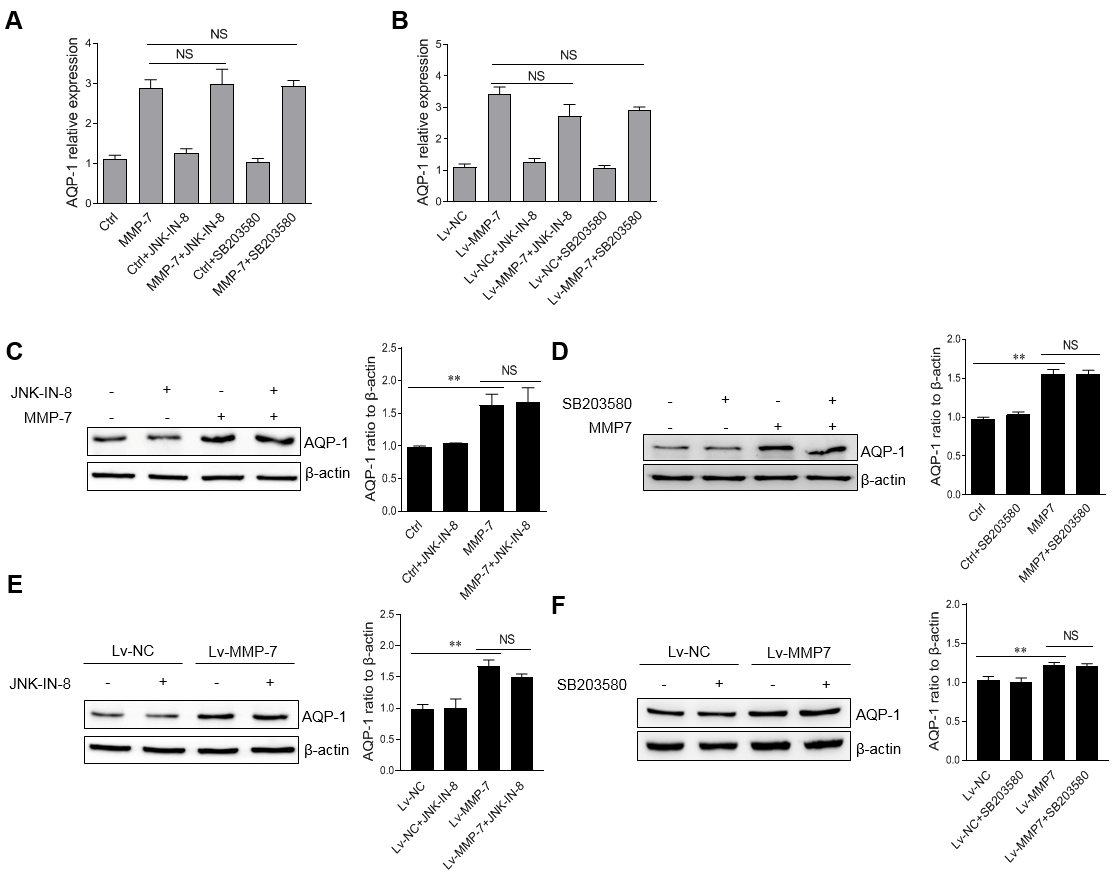


**Figure S2 Inhibition of P38 and JNK could not reverse the upregulated expression of AQP-1 by MMP-7 treatment.**

The HMrSV5 cells were seeded in the 6-well plate (1×10^6^ cells/well). (A) After pre-incubation with SB203580 for 2 hours or JNK-IN-8 for 1 hour respectively, the HMrSV5 cells were stimulated by 100ng/ml MMP- protein for 24 hours. The mRNA level of AQP-1 was detected by quantitative RT-PCR. (B) The cells were infected with MMP-7 expressing lentivirus and incubated with SB203580 or JNK-IN-8 for 24 hours. The mRNA level of AQP-1 was detected by quantitative RT-PCR. (C, D) After pre-incubation with SB203580 (C) or JNK-IN-8 (D), the HMrSV5 cells were stimulated by 100ng/ml MMP protein for another 24 hours. The protein expression of AQP-1 was detected by western blotting. (E, F) The cells were infected with MMP-7 expressing lentivirus and incubated with SB203580 (E) or JNK-IN-8 (F) for 24 hours. The protein expression of AQP-1 was detected by western blotting. ***p*<0.01, NS, not significant, one of the three independent experiments is shown.
